# Association of Peripheral Lymphocyte and the Subset Levels With the Progression and Mortality of COVID-19: A Systematic Review and Meta-Analysis

**DOI:** 10.3389/fmed.2020.558545

**Published:** 2020-09-25

**Authors:** Qun Lu, Zhengyin Wang, Yuan Yin, Yanrong Zhao, Ping Tao, Ping Zhong

**Affiliations:** ^1^Department of Laboratory Medicine, Shanghai Traditional Chinese Medicine-Integrated Hospital, Shanghai University of Traditional Chinese Medicine, Shanghai, China; ^2^Department of Infectious Diseases, Shanghai Traditional Chinese Medicine-Integrated Hospital, Shanghai University of Traditional Chinese Medicine, Shanghai, China

**Keywords:** COVID-19, lymphocytes, progression, mortality, meta-analysis

## Abstract

Current evidence is controversial in the association between peripheral lymphocyte levels and the progression and mortality of Corona Virus Disease 2019 (COVID-19), and this meta-analysis aimed to clarify the association. A systematic search was conducted in public databases to identify all relevant studies, and the study-specific odds ratio (OR) and 95% confidence intervals (CI) were pooled. Finally, 16 studies were identified with a total of 1,873 progressive COVID-19 cases and 5,177 stable COVID-19 cases. In COVID-19 progression, lymphocyte levels showed a significant negative correlation (OR: 0.68, 95% CI: 0.51–0.89), but it was not significant in the subsets of CD3+ T cells (OR: 0.97, 95% CI: 0.93–1.02), CD4+ T cells (OR: 0.93, 95% CI: 0.80–1.08), CD8+ T cells (OR: 0.96, 95% CI: 0.92–1.00), B cells (OR: 0.98, 95% CI: 0.92–1.04), or NK cells (OR: 0.80, 95% CI: 0.61–1.04). In COVID-19 mortality, lymphocyte levels showed a significant negative correlation (OR: 0.41, 95% CI: 0.20–0.85), but it was not significant in the subsets of CD3+ T cells (OR: 0.95, 95% CI: 0.86–1.05), CD4+ T cells (OR: 1.06, 95% CI: 0.86–1.31), CD8+ T cells (OR: 0.38, 95% CI: 0.14–1.01), B cells (OR: 0.98, 95% CI: 0.92–1.04), or NK cells (OR: 0.80, 95% CI: 0.61–1.04). In conclusion, current evidence suggests a significant negative association of peripheral lymphocyte levels with COVID-19 progression and mortality, but it was not significant in the subsets of CD3+ T cells, CD4+ T cells, CD8+ T cells, B cells, and NK cells.

## Introduction

In December 2019, an outbreak of pneumonia of unknown cause occurred in Wuhan, and rapidly spread throughout the world (1). The pathogen was confirmed to be a distinct clade of the β-coronavirus associated with human severe acute respiratory syndrome (SARS) (2). The novel virus was officially named SARS-CoV-2, with the disease termed COVID-19. Epidemiological data demonstrated high infectivity in SARS-CoV-2 and high mortality in multiple cohorts. Thus, it was important to identify laboratory parameters capable of discriminating the COVID-19 patients at high risk of progression or mortality, which would help physicians to provide timely intervention and improve the patients' prognosis.

Lymphocytes and the subsets of T cells, B cells, and NK cells play a key role in the maintenance of immune system function. After SARS-CoV-2 infection, the patients were characterized by a significant decrease of peripheral lymphocytes and the subsets (3). However, current studies are considered controversial on the association between peripheral lymphocyte levels at baseline and the progression and mortality of COVID-19, and no meta-analyses have focused on this. Thus, we conducted a systematic review and meta-analysis to clarify the association.

## Methods

### Literature Search

The databases of PubMed, China Wanfang Database, China Knowledge Resource Integrated Database (CNKI) were searched from inception to July 15, 2020, using key words including: (“COVID-19” OR “Corona Virus Disease 2019” OR “SARS-CoV-2” OR “2019-nCoV” OR “2019 novel coronavirus”) AND (“fatality” OR “mortality” OR “survivor” OR “non-survivor” OR “decease” OR “death” OR “prognosis” OR “progression” OR “outcome” OR “risk factor” OR “efficacy” OR “recovery”). Studies in languages other than English or Chinese were excluded. Moreover, we also reviewed the references of related studies and reviews for undetected studies. This study was approved by the ethics committee of Shanghai University of Traditional Chinese Medicine.

### Study Selection and Exclusion

We selected the studies with full texts available. The studies were included if they met the following criteria: (i) all hospitalized patients discussed had a definite diagnosis of COVID-19; (ii) the patients discussed were divided into the progressive group [e.g., admission to an intensive care unit (ICU), the use of mechanical ventilation, or death] or the stable group during the hospitalization; (iii) the study evaluated the association of the baseline lymphocytes levels or the main subtypes of CD3+ T cells, CD4+ T cells, CD8+ T cells, B cells, or NK cells (measured by multiple-color flow cytometry with human monoclonal antibodies) with the COVID-19 progression or mortality; (iv) presented relative risk (RR), odds ratio (OR), or hazard ratio (HR) estimates with 95% confidence intervals (CI). The exclusion criteria were as follows: abstracts without full texts, reviews, and case reports.

### Data Extraction and Quality Assessment

Two authors extracted the data by a standardized collection form. All differences were resolved by discussion. In each study, the following information was extracted: first author, publication year, study area, diagnostic criteria, clinical outcomes, number of cases per group, lymphocyte types, effect sizes with 95% CI, and adjusted factors. The Newcastle-Ottawa Scale (NOS) was used to assess the methodological quality of the included studies.

### Statistical Analysis

To compute a summary OR with its 95% CI, we used the study-specific most adjusted OR or HR and its 95% CI in all analyses. The heterogeneity among studies was estimated by the *Q*-test and *I*^2^ statistic. *I*^2^ > 50% represented substantial heterogeneity, and the summary estimate was analyzed by a random-effects model. Otherwise, a fixed-effects model was applied. Publication bias was assessed by using funnel plots and Egger's test. All statistical analyses were performed using the software STATA version 11.0 (StataCorp LP, College Station, TX, USA), and all tests were sided with a significance level of 0.05.

## Results

### Characteristics of the Included Studies

**Figure 1:**
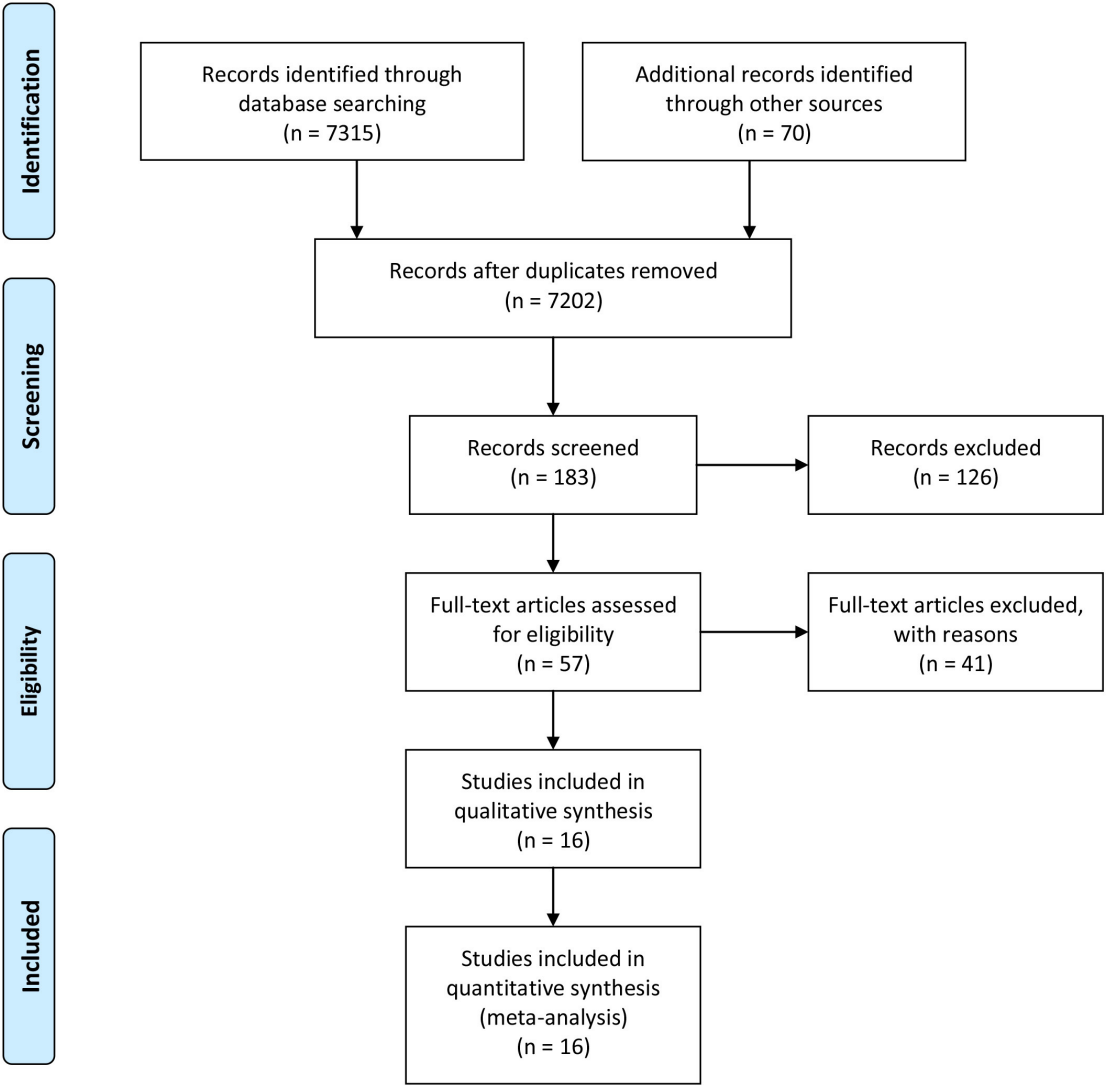
Flowchart of literature search.

The search strategy identified 7,385 records: 6,528 from PubMed, 465 from CNKI, 322 from Wangfan, and 70 from other sources (Figure 1). After excluding duplicated and irrelevant records, 16 studies were included in this meta-analysis with a total of 10,624 COVID-19 cases (Table 1) (4–19). Thirteen studies derived from China, while one study came from the USA, one from Spain and one from India. Fourteen studies were case-control designed, and the studies by Du et al. and Petrilli et al. were prospective designed. During the hospitalization, the cases were divided into the progressive group (*n* = 1,873) and the stable group (*n* = 5,177). Ten studies were adjusted by multivariable analysis. In quality assessment, the NOS scores ranged from 6 to 8, with an average of 7.13.

**Table 1:** Characteristics of included studies.

**References**	**Area**	**Diagnostic criteria**	**Clinical outcomes**	**Sample size**	**Incident cases**	**Indicators**	Effect sizes^*^	**Adjustment**
Aggarwal et al. (4)	India	WHO interim guidance	ICU admission, mechanical ventilation, death	32	12	Lymphocytes	0.47 (0.08–2.81)	-
Chen et al. (5)	China	Chinese interim guidance; WHO interim guidance	ICU admission	249	22	Lymphocytes	4.05 (0.89–18.50)	Age, male, comorbidity, WBC, CRP, albumin, AST, LDH, eGFR
						CD4+ T cells	0.55 (0.33–0.92)	
Du et al. (6)	China	WHO interim guidance	Death	179	21	Lymphocytes	0.273 (0.061–13.415)	-
						CD8+ T cells	0.251 (0.071–0.883)	Age, hypertension, cardiovascular or cerebrovascular diseases, dyspnea, fatigue, sputum production, headache, WBC, neutrophils, cTnI, myoglobin, creatinine, D-dimer, blood pressure
Guan et al. (7)	China	WHO interim guidance	ICU admission, mechanical ventilation, death	879	54	Lymphocytes	0.38 (0.13–1.06)	-
Yun et al. (8)	China	Chinese interim guidance (5th edition)	ICU admission	292	21	CD3+ T cells	0.996 (0.991–1.000)	Neutrophils, ALT, AST, albumin, LDH, creatinine, cystatin-C, transferrin, CRP, procalcitonin, D-dimer, creatine kinase, CK-MB, NT-proBNP, cTnI, myoglobin
						CD8+ T cells	1.006 (1.001–1.010)	
Liu et al. (9)	China	Chinese interim guidance (4th edition)	Disease progression, death	78	11	Lymphocytes	0.625 (0.428–65.868)	-
Liu et al. (10)	China	WHO interim guidance	Death	245	33	Lymphocytes	0.86 (0.34–2.15)	Age, sex, BMI, hypertension, chronic liver disease, HIV infection, COPD, smoking, respiratory rate, ALT, creatinine, PT, D-dimer
Luo et al. (11)	China	WHO interim guidance	Death	1,018	201	CD8+ T cells	0.169 (0.105–0.272)	Age, sex, hypertension, CHD, diabetes, pulmonary diseases
Pan et al. (12)	China	Chinese interim guidance; WHO interim guidance	Death	124	89	Lymphocytes	0.249 (0.090–7.550)	Sex, SpO2, breath rate, diastolic pressure, neutrophil, CRP, PCT, LDH, D-dimer
Petrilli et al. (13)	USA	WHO interim guidance	ICU admission, mechanical ventilation, discharge to hospice, death	2,725	990	Lymphocytes	0.57 (0.40–8.73)	Time, age, sex, race, smoking, BMI, underlying diseases, temperature, SpO2, ALT, AST, CRP, D-dimer, ferritin, PCT, TnI
			Death	2,737	424	Lymphocytes	0.72 (0.55–10.48)	
Urra et al. (14)	Spain	WHO interim guidance	ICU admission	172	27	Lymphocytes	0.769 (0.687–0.861)	-
						CD8+ T cells	0.380 (0.152–0.950)	
Wu et al. (15)	China	WHO interim guidance	ARDS	201	84	Lymphocytes	0.37 (0.21–0.63)	-
						CD3+ T cells	0.83 (0.72–0.96)	
						CD4+ T cells	0.74 (0.59–0.93)	
						CD8+ T cells	0.74 (0.53–1.04)	
			Death	84	44	Lymphocytes	0.51 (0.22–1.17)	-
						CD3+ T cells	0.81 (0.59–1.11)	
						CD4+ T cells	0.83 (0.51–1.35)	
						CD8+ T cells	0.51 (0.24–1.09)	
Xu et al. (16)	China	Chinese interim guidance (6th edition)	Death	239	147	Lymphocytes	0.81 (0.58–12.5)	Age, malignancy, platelet, ARDS, acute cardiac injury, AKI, liver dysfunction, coagulopathy
Zhang and Han (17)	China	WHO interim guidance	Death	409	102	Lymphocytes	0.012 (0.001–0.128)	Age, sex, diarrhea, WBC, neutrophil
						CD3+ T cells	0.968 (0.933–1.004)	-
						CD4+ T cells	1.114 (0.997–1.244)	
						CD8+ T cells	0.835 (0.745–0.937)	
						B cells	0.979 (0.923–1.039)	
						NK cells	0.796 (0.611–1.039)	
Zhou et al. (18)	China	WHO interim guidance	Death	191	54	Lymphocytes	0.19 (0.02–1.62)	Age, D-dimer, SOFA score, coronary heart disease
Zhou et al. (19)	China	Chinese interim guidance (5th edition)	Aggravation	17	5	Lymphocytes	0.997 (0.993–0.999)	WBC, CRP, albumin, LDH, D-dimer
						CD4+ T cells	0.995 (0.989–1.000)	
						CD8+ T cells	0.993 (0.984–1.002)	

*WHO, World Health Organization; ARDS, acute respiratory distress syndrome; WBC, white blood cells; CRP, C-reactive protein; PCT, procalcitonin; ALT, alanine aminotransferase; AST, aspartate aminotransferase; LDH, lactate dehydrogenase; eGFR, estimated glomerular filtration rate; cTnI, cardiac Troponin I; BMI, body mass index; COPD, chronic obstructive pulmonary disease; CHD, coronary heart disease; ARDS, acute respiratory distress syndrome; AKI, acute kidney injury; PT, prothrombin time; NT-proBNP, N terminal pro B type natriuretic peptide; SOFA, Sequential Organ Failure Assessment*.

* *Effect sizes for the lowest vs. highest lymphocyte levels were adjusted to the highest vs. the lowest*.

### Lymphocytes and COVID-19 Progression

**Figure 2:**
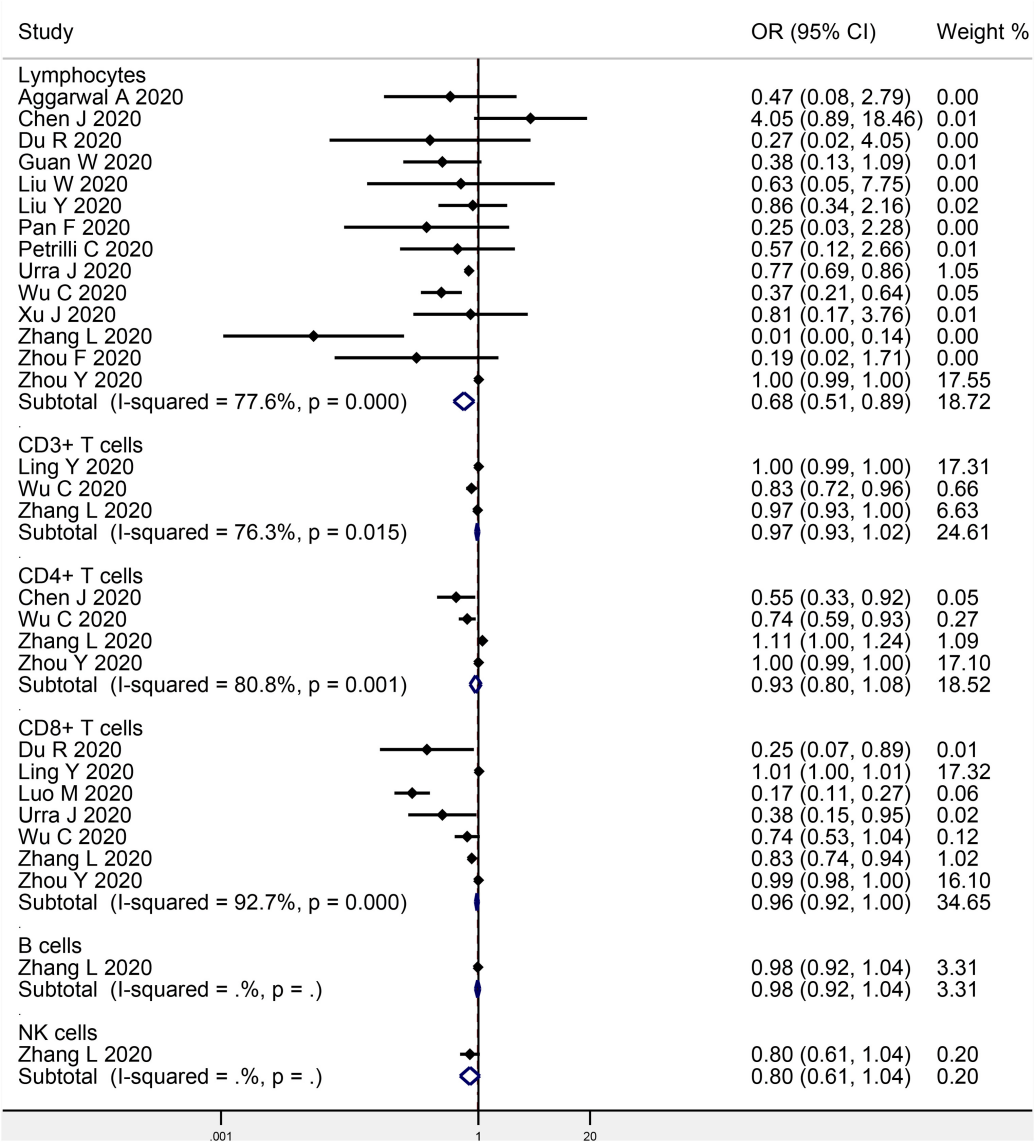
Meta-analysis of the association of peripheral lymphocytes or the subsets with COVID-19 progression.

Fourteen studies investigated the association between baseline lymphocyte levels and COVID-19 progression, with a total of 1,651 progressive cases and 4,089 stable cases (Figure 2). The meta-analysis indicated a significant negative association (OR: 0.68, 95% CI: 0.51–0.89, *P* = 0.006; *I*^2^ = 77.6%, *P* < 0.001). Egger's test detected no significant publication bias (*P* = 0.707).

### Lymphocyte Subsets and COVID-19 Progression

Three studies focused on the association between CD3+ T cells and COVID-19 progression (207 progressive cases and 695 stable cases), while four studies on CD4+ T cells (231 progressive cases and 663 stable cases), seven studies on CD8+ T cells (461 progressive cases and 1,827 stable cases), one study on B cells (102 progressive cases and 307 stable cases), and one study on NK cells (102 progressive cases and 307 stable cases) (Figure 2). The meta-analysis indicated no obvious association in CD3+ T cells (OR: 0.97, 95% CI: 0.93–1.02, *P* = 0.190; *I*^2^ = 76.3%, *P* = 0.015), CD4+ T cells (OR: 0.93, 95% CI: 0.80–1.08, *P* = 0.345; *I*^2^ = 80.8%, *P* = 0.001), CD8+ T cells (OR: 0.96, 95% CI: 0.92–1.00, *P* = 0.061; *I*^2^ = 92.7%, *P* < 0.001), B cells (OR: 0.98, 95% CI: 0.92–1.04, *P* = 0.482), or NK cells (OR: 0.80, 95% CI: 0.61–1.04, *P* = 0.092). Egger's test detected no significant publication bias in CD8+ T cells (*P* = 0.053).

### Lymphocytes and COVID-19 Mortality

**Figure 3:**
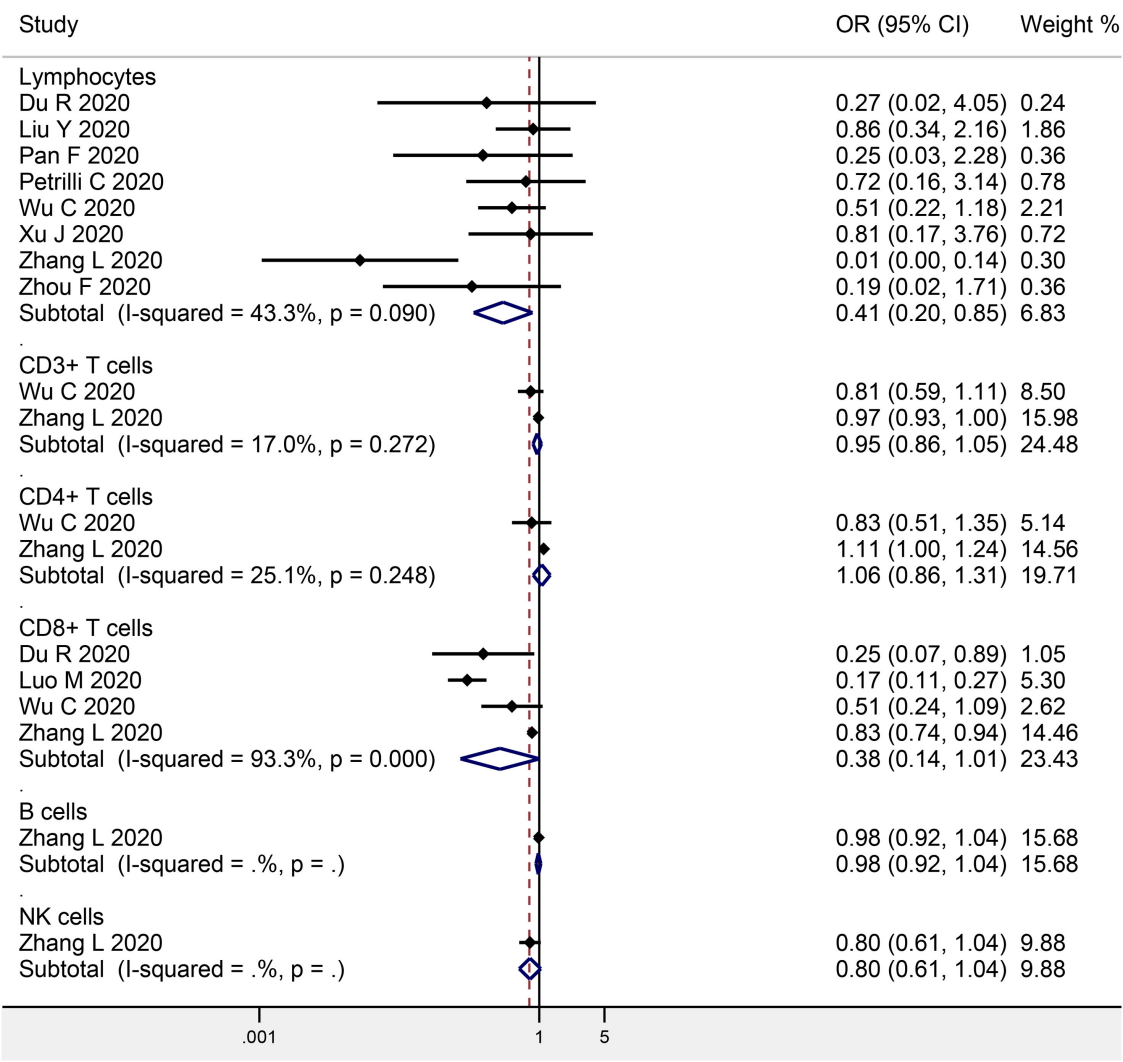
Meta-analysis of the association of peripheral lymphocytes or the subsets with COVID-19 mortality.

Eight studies investigated the association between baseline lymphocyte levels and COVID-19 mortality, with a total of 914 non-survivors and 3,294 survivors (Figure 3). The meta-analysis indicated a significant negative association (OR: 0.41, 95% CI: 0.20–0.85, *P* = 0.016; *I*^2^ = 43.3%, *P* = 0.090). Egger's test detected no significant publication bias (*P* = 0.445).

### Lymphocyte Subsets and COVID-19 Mortality

Two studies focused on the association between CD3+ T cells and COVID-19 mortality (146 non-survivors and 347 survivors), while two studies on CD4+ T cells (146 non-survivors and 347 survivors), four studies on CD8+ T cells (368 non-survivors and 1,322 survivors), one study on B cells (102 progressive cases and 307 stable cases), and one study on NK cells (102 progressive cases and 307 stable cases) (Figure 3). The meta-analysis indicated no obvious association in CD3+ T cells (OR: 0.95, 95% CI: 0.86–1.05, *P* = 0.345; *I*^2^ = 17.0%, *P* = 0.272), CD4+ T cells (OR: 1.06, 95% CI: 0.86–1.31, *P* = 0.579; *I*^2^ = 25.1%, *P* = 0.248), CD8+ T cells (OR: 0.38, 95% CI: 0.14–1.01, *P* = 0.052; *I*^2^ = 93.3%, *P* < 0.001), B cells (OR: 0.98, 95% CI: 0.92–1.04, *P* = 0.482), or NK cells (OR: 0.80, 95% CI: 0.61–1.04, *P* = 0.092).

## Discussion

As with SARS and MERS, lymphopenia was common in COVID-19 patients, suggesting an impairment of the immune system in the pathogenesis of the SARS-CoV-2 infection. In subsets, CD4+ T cells, CD8+ T cells, B cells, and NK cells were found with a decrease in COVID-19 patients (3). On admission, severe cases had a lower level of lymphocytes, CD4+ T cells, CD8+ T cells, and B cells than mild cases, which was similar in SARS (20, 21). Thus, it was thought that lymphopenia was associated with not only COVID-19 severity but also its prognosis. However, the multivariable analyses in several studies found no significant association of lymphocytes with the COVID-19 progression or mortality (4, 5, 10, 12, 13, 16, 18, 19). This aroused our attention on whether peripheral lymphocytes or the subsets could be a potential predictor for the COVID-19 prognosis.

Finally, our meta-analysis found a significant negative association of peripheral lymphocyte levels with COVID-19 progression or mortality. Lymphopenia was commonly reported in patients with COVID-19 (72%), indicating an impairment of the immune system during the course of the SARS-CoV-2 infection. This might be caused by direct attachment of SARS-CoV-2 or indirect immune injuries from inflammatory responses. On the other hand, the infiltration of peripheral lymphocytes into the inflamed lung tissues could also result in the decrease in lymphopenia. Thus, the baseline and post-treatment alteration of peripheral lymphocyte levels were thought to be reliable indicators of COVID-19 progression or mortality.

However, no subsets showed a significant association with COVID-19 progression or mortality. These findings were similar to the results in the Wang et al. study (3). In their study, the subsets of CD4+ T cells, CD8+ T cells, B cells, and NK cells decreased in COVID-19 patients, and severe cases had a lower level than mild cases. However, the baseline levels of the subsets showed no association with the clinical outcomes after one-week of treatment. Nevertheless, post-treatment decrease of CD8+ T cells and B cells and increase of CD4+/CD8+ ratio were independent predictors for poor efficacy. This might contribute to the ubiquity of lymphopenia in COVID-19, which reduced its specificity in prognostic prediction. Secondly, the patients with lower lymphocyte levels tended to have a severe infection, which caused a general inflammatory status and a more severe disease evaluation. For those patients, anti-inflammatory treatment showed a good efficacy, and the patients with a slow or meager response to the inflammation were more likely to development refractory diseases. This could also explain the findings by the Mo et al. study that the patients without fever on admission were at high risk of poor efficacy (22). Thus, it was not the baseline levels of the subsets but the post-treatment alteration that might be reliable indictors for the prediction of COVID-19 progression or mortality. On the other hand, we only investigated the subsets of T cells, CD4+ T cells, CD8+ T cells, B cells, and NK cells, and the other subsets might have an obvious change, involving naïve, central memory, effector memory, and terminally differentiated cells, as well as regulatory T cells and PD1^+^CD57^+^ exhausted T cells (23, 24).

This meta-analysis had several strengths. Firstly, to the best of our knowledge, this was the first meta-analysis to evaluate the association between peripheral lymphocyte levels and the progression and mortality of COVID-19. Secondly, we included the subsets of CD3+ T cells, CD4+ T cells, CD8+ T cells, B cells, and NK cells, and investigated not only the association with COVID-19 progression but also with the mortality. However, several limitations in this study should be considered. Firstly, the number of cases and controls in some studies was relatively small. Secondly, the obvious heterogeneity between studies was observed. Thirdly, the analyses of some subsets were limited in the number of included studies.

In conclusion, current evidence suggests a significant negative association of peripheral lymphocyte levels with COVID-19 progression and mortality, but it was not significant in the subsets of CD3+ T cells, CD4+ T cells, CD8+ T cells, B cells, and NK cells.

## Data Availability Statement

The original contributions presented in the study are included in the article/supplementary material, further inquiries can be directed to the corresponding author/s.

## Author Contributions

QL and PZ designed the study and wrote the manuscript. QL and ZW collected the data. YY, YZ, and PT analyzed the data. All authors contributed to the article and approved the submitted version.

## Conflict of Interest

The authors declare that the research was conducted in the absence of any commercial or financial relationships that could be construed as a potential conflict of interest.

## References

[1] Tu H Tu S Gao S Shao A Sheng J The epidemiological and clinical features of COVID-19 and lessons from this global infectious public health event. J Infect. (2020) 81 10.1016/j.jinf.2020.04.011 PMC7166041 32315723

[2] Rothan HA Byrareddy SN. The epidemiology and pathogenesis of coronavirus disease (COVID-19) outbreak. J Autoimmunity. (2020) 109:102433. 10.1016/j.jaut.2020.102433 32113704 PMC7127067

[3] Wang F Nie J Wang H Zhao Q Xiong Y Deng L. Characteristics of peripheral lymphocyte subset alteration in COVID-19 pneumonia. J Infect Dis. (2020) 221:1762–9. 10.1093/infdis/jiaa150 32227123 PMC7184346

[4] Aggarwal A Shrivastava A Kumar A Ali A. Clinical and epidemiological features of SARS-CoV-2 patients in SARI ward of a tertiary care Centre in New Delhi. J Assoc Phys India. (2020) 68:19–26. 32602676

[5] Chen J Qi T Liu L Ling Y Qian Z Li T. Clinical progression of patients with COVID-19 in Shanghai, China. J Infect. (2020) 80:e1–6. 10.1016/j.jinf.2020.03.004 32171869 PMC7102530

[6] Du RH Liang LR Yang CQ Wang W Cao TZ Li M. Predictors of mortality for patients with COVID-19 pneumonia caused by SARS-CoV-2: a prospective cohort study. Eur Respir J. (2020) 55:2000524. 10.1183/13993003.00524-2020 32269088 PMC7144257

[7] Guan WJ Ni ZY Hu Y Liang WH Ou CQ He JX Clinical characteristics of coronavirus disease 2019 in China. N Engl J Med. (2020) 382:1708–20. 10.1056/NEJMoa2002032 32109013 PMC7092819

[8] Yun L Yixiao L Zhiping Q Dan H Dandan Z Tao L Clinical analysis of risk factors for severe patients with novel coronavirus pneumonia. Chinese Med J Netw. (2020) 38 10.3760/cma.j.cn311365-20200211-00055

[9] Liu W Tao ZW Lei W Ming-Li Y Kui L Ling Z. Analysis of factors associated with disease outcomes in hospitalized patients with 2019 novel coronavirus disease. Chinese Med J. (2020) 133:1032–8. 10.1097/CM9.0000000000000775 32118640 PMC7147279

[10] Liu Y Du X Chen J Jin Y Peng L Wang HHX. Neutrophil-to-lymphocyte ratio as an independent risk factor for mortality in hospitalized patients with COVID-19. J Infect. (2020) 81:e6–12. 10.1016/j.jinf.2020.04.002 32283162 PMC7195072

[11] Luo M Liu J Jiang W Yue S Liu H Wei S. IL-6 and CD8+ T cell counts combined are an early predictor of in-hospital mortality of patients with COVID-19. JCI Insight. (2020) 5:139024. 10.1172/jci.insight.139024 32544099 PMC7406244

[12] Pan F Yang L Li Y Liang B Li L Ye T. Factors associated with death outcome in patients with severe coronavirus disease-19 (COVID-19): a case-control study. Int J Med Sci. (2020) 17:1281–92. 10.7150/ijms.46614 32547323 PMC7294915

[13] Petrilli CM Jones SA Yang J Rajagopalan H O'Donnell L Chernyak Y. Factors associated with hospital admission and critical illness among 5,279 people with coronavirus disease 2019 in New York City: prospective cohort study. (2020) 369:m1966. 10.1136/bmj.m1966 32444366 PMC7243801

[14] Urra JM Cabrera CM Porras L Ródenas I. Selective CD8 cell reduction by SARS-CoV-2 is associated with a worse prognosis and systemic inflammation in COVID-19 patients. Clin Immunol. (2020) 217:108486. 10.1016/j.clim.2020.108486 32479985 PMC7256549

[15] Wu C Chen X Cai Y Xia J Zhou X Xu S. Risk factors associated with acute respiratory distress syndrome and death in patients with coronavirus disease 2019 pneumonia in Wuhan, China. JAMA Internal Med. (2020) 180:1–11. 10.1001/jamainternmed.2020.0994 32167524 PMC7070509

[16] Xu J Yang X Yang L Zou X Wang Y Wu Y. Clinical course and predictors of 60-days mortality in 239 critically ill patients with COVID-19: a multicenter retrospective study from Wuhan, China. (2020) 24:394. 10.1186/s13054-020-03098-9 32631393 PMC7336107

[17] Zhang L Han C. Diarrhea and altered inflammatory cytokine pattern in severe coronavirus disease 2019: impact on disease course and in-hospital mortality. J Gastroenterol Hepatol. (2020). 10.1111/jgh.15166 32602128

[18] Zhou F Yu T Du R Fan G Liu Y Liu Z Clinical course and risk factors for mortality of adult inpatients with COVID-19 in Wuhan, China: a retrospective cohort study. Lancet. (2020) 395:1054–62. 10.1016/S0140-6736(20)30566-3 32171076 PMC7270627

[19] Zhou Y Zhang Z Tian J Xiong S. Risk factors associated with disease progression in a cohort of patients infected with the 2019 novel coronavirus. Ann Palliative Med. (2020) 9:428–36. 10.21037/apm.2020.03.26 32233642

[20] Wong RS Wu A To KF Lee N Lam CW Wong CK. Haematological manifestations in patients with severe acute respiratory syndrome: retrospective analysis. BMJ. (2003) 326:1358–62. 10.1136/bmj.326.7403.1358 12816821 PMC162124

[21] Peiris JS Lai ST Poon LL Guan Y Yam LY Lim W. Coronavirus as a possible cause of severe acute respiratory syndrome. Lancet. (2003) 361:1319–25. 10.1016/S0140-6736(03)13077-2 12711465 PMC7112372

[22] Mo P Xing Y Xiao Y Deng L Zhao Q Wang H. Clinical characteristics of refractory COVID-19 pneumonia in Wuhan, China. Clin Infect Dis. (2020) ciaa270. 10.1093/cid/ciaa270 32173725 PMC7184444

[23] De Biasi S Meschiari M Gibellini L Bellinazzi C Borella R Fidanza L. Marked T cell activation, senescence, exhaustion and skewing towards TH17 in patients with COVID-19 pneumonia. Nat Commun. (2020) 11:3434. 10.21203/rs.3.rs-23957/v1 32632085 PMC7338513

[24] Weiskopf D Schmitz KS. Phenotype and kinetics of SARS-CoV-2-specific T cells in COVID-19 patients with acute respiratory distress syndrome. Sci Immunol. (2020) 5:abd2071. 10.1126/sciimmunol.abd2071 32591408 PMC7319493

